# Effect of Dietary Methylsulfonylmethane Supplementation on Growth Performance, Hair Quality, Fecal Microbiota, and Metabolome in Ragdoll Kittens

**DOI:** 10.3389/fmicb.2022.838164

**Published:** 2022-07-04

**Authors:** Dan Guo, Limeng Zhang, Lingna Zhang, Sufang Han, Kang Yang, Xinye Lin, Chaoyu Wen, Aorigeile Tong, Meiyu Zhang, Yulong Yin, Baichuan Deng

**Affiliations:** ^1^Guangdong Laboratory for Lingnan Modern Agriculture, Guangdong Provincial Key Laboratory of Animal Nutrition Control, National Engineering Research Center for Breeding Swine Industry, College of Animal Science, South China Agricultural University, Guangzhou, China; ^2^Guangzhou Qingke Biotechnology Co., Ltd., Guangzhou, China; ^3^College of Animal Science and Technology, Guangdong Polytechnic of Science and Trade, Guangzhou, China; ^4^Key Laboratory of Agro-Ecological Processes in Subtropical Region, Institute of Subtropical Agriculture, Chinese Academy of Sciences, Changsha, China

**Keywords:** methylsulfonylmethane, feline nutrition, pet nutrition, kittens, cats, hair quality, fecal microbiota, metabolism

## Abstract

Methylsulfonylmethane (MSM) is a natural sulfur-containing organic substance that has many biological functions, such as antioxidant, anti-inflammatory, skin nourishing, and hair growth-promoting effects. This study was conducted to determine the effect of MSM supplementation on growth performance, antioxidant capacity, and hair quality in kittens. A total of 21 Ragdoll kittens were assigned to three diets by initial body weight and gender: basal diet supplemented with 0%, 0.2%, and 0.4% MSM (CON, LMSM, and HMSM groups) for 65 days. During the whole period, the food intake of kittens in the MSM-treated groups tended to be higher (*P* < 0.10) compared with the CON group, and the average daily gain (ADG) had no significant difference when compared to the kittens in the CON group (*P* > 0.05). Antioxidant capacity had no significant difference (*P* > 0.05) among the groups. The scale thickness of hair tended to be smaller in the LMSM group compared to the CON group (*P* < 0.10) and decreased significantly (*P* < 0.05) over time from d 0 to d 65 in the LMSM group, indicating the improvement of hair quality. Besides, supplementation with LMSM increased bacterial diversity. Kittens fed MSM had no significant differences in fecal genus at the end of the study. No significant differences in fecal short-chain fatty acids were observed among groups. Fecal metabolomics analysis further revealed that MSM hardly affected the metabolites. Overall, dietary supplementation with 0.2% MSM can improve the hair quality of kittens. Furthermore, 0.2∼0.4% of MSM had no detrimental effects on serum biochemistry, growth performance, gut microbiota, and metabolome, which supports the safety inclusion of MSM to a certain degree in feline diets. To the best of our knowledge, this is the first study to investigate the effects of MSM supplementation in cats.

## Introduction

Nowadays, pets (mainly cats and dogs) are cared for and raised as companion animals to provide humans social support and companionship (Staats et al., 2008). The rise of companion animal ownership is accompanied by the growth of economics and the improvement of material conditions (Shepherd, 2008). According to recent reports, the global feline population has been rising steadily, with household cat numbers reaching approximately 1.1 billion in Europe (Statista, 2021b), 95.6 million in the United States (Statista, 2021a), and 48.6 million in China (Petfoodindustry, 2021). Pet nutrition differs greatly from traditional livestock and poultry nutrition in that economic benefits are not the priority consideration. Rather, animal appearance, physical/mental health, and longevity are receiving more and more attention in the pet nutrition field (Buchanan et al., 2011). Due to ornamental attributes, outstanding hair quality can improve pets’ appearance. Therefore, maintenance of the health of pet hair is considered an important aspect of pet ownership and draws great attention from pet owners.

Methylsulfonylmethane (MSM or dimethylsulfone) is a stable metabolite of dimethylsulfoxide (DMSO) that is part of the sulfur cycle in nature and is widely present in different organisms, including animals and humans (Richmond, 1986). A variety of health-specific measures are improved with MSM supplementation, such as anti-inflammation (Kim et al., 2009), antioxidant (Beilke et al., 1987), and anti-tumor activities (Pn et al., 2015; Karabay et al., 2016; Kowalska et al., 2021). Recent evidence has found that MSM exhibits bacteriostatic inhibition of *Escherichia coli* and *Salmonella enterica* Kinshasa *in vitro* (Poole et al., 2019). However, there are few studies on the effects of MSM on gut microbiota and metabolomics *in vivo*. Toxicity studies have supported the safe use of MSM (Magnuson et al., 2007; Abdul et al., 2019). The United States Food and Drug Administration refers to MSM as a natural source of sulfur in the skin, hair, nails, and egg whites that can be used as a joint health supplement (U. S. Food and Drug Administration, 2004). Evidence for the growth-promoting function of MSM is also available. For example, MSM can nourish human skin (Muizzuddin and Benjamin, 2020), promote the growth performance of growing-finishing pigs (Ho et al., 2006), and stimulate hair growth in mice (Shanmugam et al., 2009).

Even though studies have investigated the effects of MSM in ducks, rodents, and other animals, its application in domestic cats has not been addressed. Therefore, the objective of this study is to determine the effects of MSM as a feed additive on growth performance, antioxidant capacity, hair quality, fecal microbiota, and metabolome in kittens.

## Materials and Methods

### Animal Ethics

All experimental procedures were authorized by the Animal Care and Use Committee prior to animal experimentation (Approval number: 2021a030) and were performed following the guidelines of the Laboratory Animal Center at the South China Agricultural University.

### Experimental Design, Animals, and Management

Twenty-one Ragdoll kittens, 7 males and 14 females, with a mean body weight (BW) of 3.42 ± 0.29 kg and age 6–8 months were included. The study lasted for 65 days, including a 5-day wash-out period and a 60-day experimental period. Kittens were allotted to one of the three treatment groups (*n* = 7/group) by initial body weight and the gender: fed control (CON; 0% MSM in diet), low dose (LMSM; 0.2% MSM in diet), and high dose (HMSM; 0.4% MSM in diet) diet, respectively. The mean baseline BW of kittens showed no significant difference among the groups (*P* > 0.05). Fresh adequate food was offered at 8:30 a.m. on the daily basis to carry out free-choice feeding. Daily food intake (g/day) was recorded for each kitten. The scale of feeding was in the range of 100–130 g. The vaccination and deworming treatments were conducted prior to the study, and kittens did not receive any drugs that can cause changes to the intestinal microflora (e.g., antibiotics) within 1 month prior to the study. Kittens were housed individually in cages (1.1 × 0.7 × 0.6 m^3^) and were able to socialize and exercise outside of their cages in a room for about 1 h with temperature maintained at 23–25°C and humidity at 40–60%. Kittens were allowed to access various toys and scratch poles for environmental enrichment and to interact with humans. Freshwater was available *ad libitum* in bowls. Cages were cleaned and disinfected termly.

### Diet

The details of the chemical and energy composition of the basal diet is given in Table 1. The extruded dry diet was manufactured at Guangzhou Qingke Biotechnology Co., Ltd., and formulated to meet the nutrient recommendations of the Association of American Feed Control Officials (Association of American Feed Control Officials, 2017) for kittens (AOAC, 2000). The three experimental diets had the same ingredient composition except for the MSM inclusion.

**TABLE 1:** Experimental diet composition and nutrients.

Items	Diets
**Ingredients (as-is basis,%)**	
Enzymatic meat	24.00
Chicken meat meal	40.90
Sweet potato flour	6.00
Potato flour	5.20
Chicken oil	6.70
Solid flavoring agent	4.30
Cheese powder	0.70
Alfalfa granule	2.00
Vitamins and minerals premix ^1^	2.10
Fish oil	1.20
Whole egg powder	2.50
Kelp powder	0.60
Pumpkin seeds	0.50
Chicken liver powder	0.90
Soy Lecithin	1.00
Beer yeast	1.00
Plantago	0.20
Madder	0.20
**Analytical composition (DM basis,%)**	
DM	91.91
CP	42.34
Fat	16.57
Ash	9.02
GE, kJ/g	20.64

*^1^Provided per kilogram diet: vitamin A, 22,600.00 IU; vitamin D, 3,500.00 IU; vitamin E, 54.00 mg; vitamin K3, 0.10 mg; vitamin B1, 16.80 mg; vitamin B2, 7.40 mg; vitamin B6, 8.40 mg; vitamin B12, 0.03 mg; nicotinic acid, 98.00 mg; calcium pantothenate, 9.48 mg; D-biotin, 0.11 mg; folic acid, 0.90 mg; choline chloride, 2641.80 mg; Fe, 80.00 mg; Cu, 15.00 mg; Mn, 7.80 mg; Zn, 75.20 mg; I, 1.80 mg; and Se, 0.30 mg.*

*DM, dry matter; CP, Crude protein; Fat, Crude fat; GE, Gross energy.*

Throughout the trial period, a 200-g basal diet was collected weekly and was kept in the refrigerator at −20°C. Feed samples were dried in the oven and were ground through a 1-mm screen for chemical composition analysis. The dry matter (DM) and organic matter (OM) were determined for the diets according to AOAC (2000; method 950.46 for water and method 942.05 for crude ash) (AOAC, 2000). Acid-hydrolyzed fat was analyzed by a fatty analyzer (FT640, Guangzhou, Grand Analytical Instrument Co., Ltd.) according to AOAC (2000; method 920.39 for ether extract) (AOAC, 2000). The crude protein (CP) was determined by using the Kjeldahl method with a semi-automatic Kjeldahl apparatus (VAPODEST 200, C. Gerhardt GmbH and Co., KG, Germany) and following the Official Method of AOAC (2000; method 954.01 for crude protein) (AOAC, 2000). Diet was analyzed for GE by oxygen bomb calorimeter (IKA C 200, IKA (Guangzhou) Instrument Equipment Co., Ltd., Guangzhou, China).

### Sample Collection

#### Blood Collection and Analyses

On d 0, d 35, and d 65 after overnight fasting, 3 mL of blood samples was collected from each kitten from the forelimb vein, blood was transferred to a pre-cooled serum separator tube, and left to stand for 30 min before centrifugation at 3,500 × *g* at room temperature for 15 min. After centrifugation, the supernatants were aliquoted into microcentrifuge tubes and stored at −80°C for further analysis. Serum glucose (GLU), aspartate aminotransferase (AST), alanine aminotransferase (ALT), total protein (TP), albumin (ALB), alkaline phosphatase (ALP), urea nitrogen (BUN), creatinine (CREA), cholesterol (CHO), and triglycerides (TG) were measured using commercial kits according to biochemistry autoanalyzer (Rayto Life and Analytical Sciences Co., Ltd., Shenzhen, China). Serum antioxidant capacity was detected using commercial kits (Nanjing Jiancheng Bioengineering Institute, Nanjing, China) according to the manufacturer’s protocol, including those for superoxide dismutase (SOD), total antioxidative capacity (T-AOC), malondialdehyde (MDA), glutathione peroxidase (GSH-PX), and catalase (CAT).

#### Hair Collection and Analyses

**FIGURE 1:**
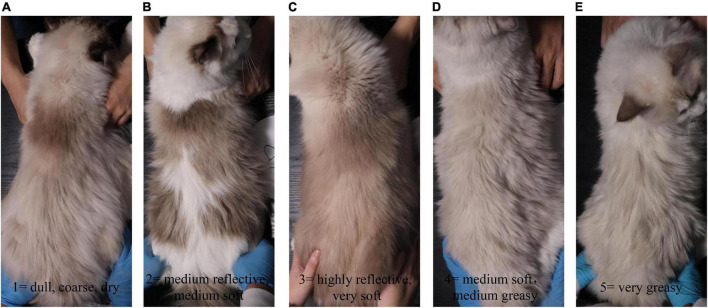
The hair rating criteria of kittens fed MSM-supplemented diets. All samples were scored in 5 increments using the following scale: **(A)** 1 = dull, coarse, dry; **(B)** 2 = medium reflective, medium soft; **(C)** 3 = highly reflective, very soft; **(D)** 4 = medium soft, medium greasy; and **(E)** 5 = very greasy.

Hair samples were collected on d 0, d 35, and d 65 for measurement of length and scale structure, the content of element, sulfur-containing amino acids, and keratin. The hair on the back of all kittens was shaved on d 0, and the length of the new hair at the same position was observed on d 35 and d 65. The hair scoring was conducted on d 0, d 35, and d 65 under the same lighting conditions and on the same observation table. The five observers were blinded to the treatment individual cat received. The method described by Rees et al. (2001) was adopted for the evaluation of hair scores (Rees et al., 2001). Briefly, all samples were scored in 5 increments using the following scale: 1 = dull, coarse, dry; 2 = medium reflective, medium soft; 3 = highly reflective, very soft; 4 = medium soft, medium greasy; and 5 = very greasy, where 1 ≤ HS ≤ 2 dry, 2 < HS < 4 ideal, and 4 ≤ HS ≤ 5 greasy (Figure 1). Hair samples were measured for the structural characteristics of the scale layer, including thickness, the height of the scale, and diameter of the hair by scanning electron microscope (SEM, JSM-6380LV, JEOL Ltd.). Because the amount of hair collected was insufficient, six repetitions were performed in each group. In addition, three samples were randomly selected from each group for the measurement of keratin, amino acids, especially the sulfur-containing amino acids, and content of S, O, and C by semi-quantitative detection analysis using an X-ray energy spectrometer (Genesis 2000, EDAX Inc.).

#### Fecal Sample Collection and Analyses

A fresh fecal sample (within 15 min of defecation) per cat was collected on d 0, d 35, and d 65 for microbiota, short-chain fatty acids (SCFAs), and metabolome analysis. A few aliquots were transferred to a 5 mL sterile fecal collection tube (BIORISE) for microbiota measurement, frozen immediately on dry ice, and stored at −80°C until DNA extraction. Another aliquot for SCFAs measurement was stored at −80°C until analysis. Finally, an aliquot was frozen immediately on dry ice and stored at −80°C until analysis prior to metabolomics analysis.

Genomic DNA of fecal samples was extracted using the cetyltrimethylammonium bromide method and was used for 16S rRNA sequencing. In short, a targeted PCR-based sequencing approach was used, where the V3–V4 regions of the 16S rRNA gene were targeted to generate amplicons using the primers 341F (5′-CCTAYGGGRBGCASCAG-3′) and 806R (5′-GGACTACNNGGGTATCTAAT-3′) with the barcode. Sequencing libraries were generated using the TruSeq^®^ DNA PCR-Free Sample Preparation Kit (Illumina, United States) following the manufacturer’s recommendations, and index codes were added. The library quality was assessed on the Qubit@ 2.0 Fluorometer (Thermo Scientific) and Agilent Bioanalyzer 2100 system. At last, the library was sequenced on an Illumina NovaSeq platform and 250-bp paired-end reads were generated. Then, reads were filtered by QIIME quality filters (QIIME1.9.1) (Caporaso et al., 2010).

Fecal SCFAs concentrations were determined by gas chromatography-mass spectrometer (GC-MS) using the GCMS-QP2020 system (Shimadzu, Tokyo, Japan). The GC was equipped with an autoinjector AOC-20i (Shimadzu) and coupled to a flame ionization detector. The chromatographic separation was performed on a DB-FFAP capillary column (30 m × 0.25 mm × 0.25 μm). The sample (0.6 μL) was injected with a 30: 1 split ratio using an autosampler. The injection port was set to a temperature of 250°C. The initial temperature of the column was 80°C for 2 min, increased to 150°C at a rate of 10°C/min for 2 min, and to 180°C at a rate of 15°C/min for 5 min. The total run time was 18 min. Helium (He, 99.999%) was the carrier gas with a flow rate of 3 mL/min. The MS parameters were electron impact mode at ionization energy of 70 eV. The ion source and interface temperatures were 230°C and 250°C, respectively. The solvent delay time was 1 min, 230°C. The acquisition mode was selected at ion monitoring mode with a scan interval of 0.3 s.

The fecal metabolome was assessed using an untargeted approach *via* liquid chromatography-mass spectrometry (Thermo Fisher Scientific). The Compound Discoverer 2.1 (Thermo Fisher Scientific) data analysis tool was employed to automate complete raw data pre-processing and was applied to identify metabolites by searching the mzCloud library and mzVault library. In this study, MetaboAnalyst 5.0^1^ was used to perform a multivariate analysis. Peak height data of metabolome were obtained and uploaded to MetaboAnalyst 5.0. Principal component analysis (PCA) of metabolites was performed. Pathway enrichment analysis was performed by using the enrichment analysis module on MetaboAnalyst 5.0. The visualization results of the models were obtained with MetaboAnalyst 5.0.

Due to the insufficient amount of fecal samples collected for some kittens, there were seven repetitions in the CON group and six repetitions in the LMSM and HMSM groups on d 0, and seven repetitions in the CON and LMSM groups and six repetitions in the HMSM group on d 35 and d 65, respectively.

### Statistical Analysis

SPSS 26.0 and GraphPad Prism 8.0 software were used for statistical analysis and graphical presentation. One-way analysis of variance (ANOVA) followed by a least-significant difference (LSD) multiple range test was used to determine the statistical significance of multiple comparisons in the experiment. Repeated measures ANOVA was employed to analyze the data over three time points. For gut microbiota, operational taxonomy units (OTUs) with similarity ≥ 97% were chosen for the α-diversity analysis, and the principal coordinate analysis (PCoA) and box plot were selected to evaluate the β-diversity. Microbiota-based biomarker discoveries were done with Student’s *t*-test and adjusted using the Benjamini–Hochberg test to get Q-values. Clustered heatmap with the rank abundance plot of bacterial genera was plotted at https://magic.novogene.com. To preliminarily screen the differential metabolites, we compared the metabolites in the MSM groups with the CON group (*P* < 0.05, FC > 2), and the *P*-values were adjusted by FDR (FDR < 0.1). The advanced volcano plot was constructed using the OmicStudio tools at https://www.omicstudio.cn/tool. Variability in the data was expressed as the standard error of the mean. Significance was considered with a *P* < 0.05 and tendency at 0.05 ≤ *P* < 0.10.

## Results

### Performance

All kittens remained healthy and showed good appetite throughout the study. Signs of gastrointestinal intolerance (e.g., diarrhea and vomit) were not observed in the subjects. Both initial (d 0) and final BW (d 65) did not differ among the three treatment groups (*P* > 0.05; Table 2). Data for the whole period showed that the food intake (FI) of kittens in the LMSM group tended to be higher than that in the CON group (*P* < 0.10), and the average daily gain (ADG) of kittens in the LMSM and HMSM groups had no significant difference when compared to the kittens in the CON group (*P* > 0.05). Treatment effects on the FI were observed during the period from d 36 to d 65 (*P* < 0.05). The FI of kittens in the LMSM and HMSM groups was significantly higher when compared to the kittens in the CON group (*P* < 0.05). There were no differences (*P* > 0.05) in the FI and ADG among treatments during the period from d 0 to d 35.

**TABLE 2:** Initial and final BW, FI, and ADG of kittens fed MSM-supplemented diets.

Items	CON	LMSM	HMSM	SEM	*P*-value
Initial BW, kg	3.43	3.62	3.36	0.27	0.927
Final BW, kg	3.46	3.92	3.62	0.22	0.700
**D 0 to d 35**					
ADG, g/d	2.16	6.50	7.51	2.58	0.690
FI, g/d	56.05	59.35	57.88	2.31	0.375
**D 35 to d 65**					
ADG, g/d	−1.24	3.52	0.86	1.74	0.559
FI, g/d	51.69*^b^*	67.48^a^	66.15^a^	2.56	0.011
**D 0 to d 65**					
ADG, g/d	0.46	5.01	4.18	1.85	0.589
FI, g/d	53.87	63.42	62.02	2.13	0.061

*^a,b^Values in a row with no common superscripts differ significantly (P < 0.05).*

*Mean values are based on one kitten per replicate and seven replicates per treatment.*

*CON, control diet (0% MSM in diet); LMSM, low dose (0.2% MSM in diet); HMSM, high dose (0.4% MSM in diet); BW, body weight; FI, food intake; ADG, average daily gain.*

### Serum Biochemistry and Antioxidant Capacity

Serum biochemistry of kittens fed diets containing MSM was within the reference range for healthy kittens and was not affected (*P* > 0.05) by dietary treatments at different time points (Table 3). Likewise, the results of antioxidant capacity were normal for all kittens and did not differ (*P* > 0.05) among the treatment groups (Table 4).

**TABLE 3:** Serum biochemistry of kittens fed MSM-supplemented diets.

Items	CON	LMSM	HMSM	SEM	*P*-value
**D 0**					
ALT, U/L	60.62	55.91	80.00	5.44	0.161
AST, U/L	33.65	35.10	40.49	2.09	0.395
ALB, g/L	37.92	36.02	36.89	0.59	0.449
TP, g/L	82.12	80.39	79.54	1.61	0.820
CREA, mol/L	102.28	100.64	96.92	3.55	0.837
TG, mmol/L	0.69	0.67	0.55	0.06	0.583
CHO, mmol/L	3.43	3.75	3.59	0.15	0.720
ALP, U/L	63.26	76.27	92.61	13.79	0.710
GLU, mmol/L	4.33	5.15	4.70	0.19	0.240
BUN, mg/dL	26.50	25.63	25.23	0.76	0.804
**D 35**					
ALT, U/L	54.98	64.61	70.77	4.39	0.355
AST, U/L	30.33	39.67	38.41	2.54	0.280
ALB, g/L	36.35	35.75	38.14	0.74	0.412
TP, g/L	69.25	75.89	75.76	1.68	0.187
CREA, mol/L	94.03	84.13	87.74	4.58	0.698
TG, mmol/L	0.49	0.45	0.46	0.04	0.926
CHO, mmol/L	3.84	3.62	4.16	0.17	0.467
ALP, U/L	58.87	68.89	68.37	6.92	0.820
GLU, mmol/L	3.50	3.25	3.31	0.10	0.578
BUN, mg/dL	28.96	30.04	27.75	0.80	0.531
**D 65**					
ALT, U/L	55.27	62.90	63.52	2.86	0.450
AST, U/L	30.94	34.50	44.95	4.53	0.448
ALB, g/L	36.68	37.17	38.22	0.78	0.736
TP, g/L	81.53	83.13	77.88	1.88	0.535
CREA, mol/L	98.51	91.10	96.32	5.45	0.865
TG, mmol/L	0.85	0.73	0.73	0.07	0.737
CHO, mmol/L	3.44	3.85	4.01	0.25	0.648
ALP, U/L	55.37	69.90	62.72	6.20	0.660
GLU, mmol/L	3.87	3.51	3.53	0.08	0.144
BUN, mg/dL	27.79	28.86	30.40	1.21	0.703

*Mean values are based on one kitten per replicate and six replicates per treatment. CON, control diet (0% MSM in diet); LMSM, low dose (0.2% MSM in diet); HMSM, high dose (0.4% MSM in diet); ALT, alanine aminotransferase; AST, aspartate aminotransferase; ALB, albumin; TP, total protein; CREA, creatinine; TG, triglycerides; CHO, cholesterol; ALP, alkaline phosphatase; GLU, glucose; BUN, urea nitrogen.*

**TABLE 4:** Serum antioxidant of kittens fed MSM-supplemented diets.

Items	CON	LMSM	HMSM	SEM	*P*-value
**D 0**					
SOD, U/mL	98.09	93.46	103.80	2.82	0.344
MDA, nmol/mL	4.56	4.56	4.78	0.17	0.851
GSH-PX, U	960.84	1289.47	1205.47	68.29	0.121
CAT, U/mL	3.31	4.83	2.22	1.06	0.626
T-AOC, mM	0.24	0.22	0.23	0.01	0.835
**D 35**					
SOD, U/mL	107.34	111.06	103.31	2.27	0.401
MDA, nmol/mL	4.17	3.95	4.11	0.12	0.778
GSH-PX, U	1118.53	1226.11	1215.79	70.40	0.809
CAT, U/mL	2.05	2.66	1.78	0.35	0.607
T-AOC, mM	0.20	0.22	0.22	0.01	0.422
**D 65**					
SOD, U/mL	102.95	104.51	98.81	2.61	0.680
MDA, nmol/mL	4.11	4.00	4.11	0.17	0.960
GSH-PX, U	1398.53	1310.64	1328.51	96.64	0.934
CAT, U/mL	1.41	3.23	2.28	0.51	0.374
T-AOC, mM	0.23	0.21	0.21	0.01	0.199

*Mean values are based on one kitten per replicate and six replicates per treatment. CON, control diet (0% MSM in diet); LMSM, low dose (0.2% MSM in diet); HMSM, high dose (0.4% MSM in diet); SOD, superoxide dismutase; MDA, malondialdehyde; GSH-PX, glutathione peroxidase; CAT, catalase; T-AOC, total antioxidative capacity.*

### The Hair Growth and Quality

There was no significant difference in the hair length of kittens in each group (*P* > 0.05). The HMSM group showed a better effect on the growth of hair at d 0 and d 35 compared to other groups, but the hair growth of the LMSM group at d 36 and d 65 was faster than other groups, as shown in Table 5.

**TABLE 5:** The length (mm) of hair from kittens fed MSM-supplemented diets.

Items	CON	LMSM	HMSM	SEM	*P*-value
D 0	0.00	0.00	0.00	-	-
D 35	18.83	17.17	22.33	2.23	0.655
D 65	23.83	25.00	24.67	2.10	0.976

*Mean values are based on one kitten per replicate and six replicates per treatment. CON, control diet (0% MSM in diet); LMSM, low dose (0.2% MSM in diet); HMSM, high dose (0.4% MSM in diet).*

Hair condition did not differ (*P* > 0.05) among treatments during the experiment, with all groups being scored in a state described as a medium level (Table 6). However, the ideal rate of hair in the LMSM group (52.38%) was higher than the CON (47.62%) and HMSM groups (42.86%), and the greasy rate of hair in the LMSM group (9.52%) was lower than the CON (23.81%) and HMSM groups (19.05%).

**TABLE 6:** Hair score of kittens fed MSM-supplemented diets.

Items	CON	LMSM	HMSM	SEM	*P*-value
Score	2.95	2.71	2.81	0.13	0.782
Ideal rate	47.62%	52.38%	42.86%	-	-
Greasy rate	23.81%	9.52%	19.05%	-	-
Drying rate	28.57%	38.10%	38.10%	-	-

*Mean values are based on one kitten per replicate and seven replicates per treatment.*

*CON, control diet (0% MSM in diet); LMSM, low dose (0.2% MSM in diet); HMSM, high dose (0.4% MSM in diet).*

**FIGURE 2:**
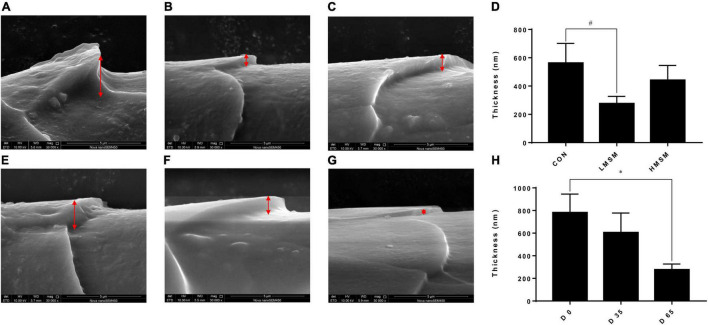
The scale thickness of hair of kittens fed MSM-supplemented diets. **(A)** The scale thickness of hair in CON, **(B)** LMSM, and **(C)** HMSM on d 65. **(D)** The scale thickness of hair from kittens fed diets containing MSM on d 65 among three groups. **(E)** The scale thickness of hair in LMSM on d 0, **(F)** d 35, and **(G)** d 65. **(H)** The scale thickness of hair from kittens fed diets containing MSM in LMSM on d 0, d 35, and d 65. CON = control diet (0% MSM in diet); LMSM = low dose (0.2% MSM in diet); and HMSM = high dose (0.4% MSM in diet). The symbol (*) indicates statistically significant differences between two groups (*P < 0.05), and the symbol (#) represents difference tendency (# 0.05 ≤ *P* < 0.10). Mean values are based on one kitten per replicate and six replicates per treatment.

**FIGURE 3:**
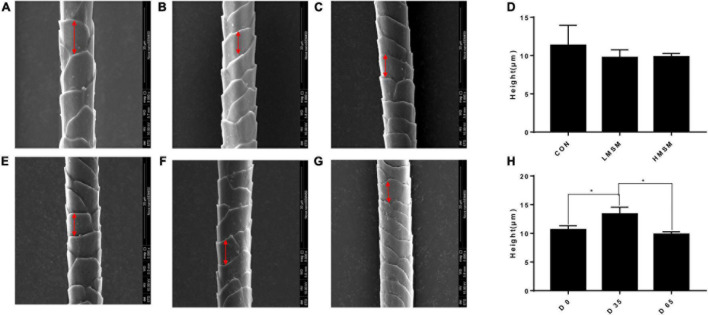
The scale height of hair of kittens fed MSM-supplemented diets. **(A)** The scale height of hair in CON, **(B)** LMSM, and **(C)** HMSM on d 65. **(D)** The scale height of hair from kittens fed diets containing MSM on d 65 among three groups. **(E)** The height of hair in HMSM on d 0, **(F)** d 35, and **(G)** d 65. **(H)** The scale height of hair from kittens fed diets containing MSM in HMSM on d 0, d 35, and d 65. CON = control diet (0% MSM in diet); LMSM = low dose (0.2% MSM in diet); HMSM = high dose (0.4% MSM in diet). The symbol (*) indicates statistically significant differences between two groups (*P < 0.05). Mean values are based on one kitten per replicate and six replicates per treatment.

**FIGURE 4:**
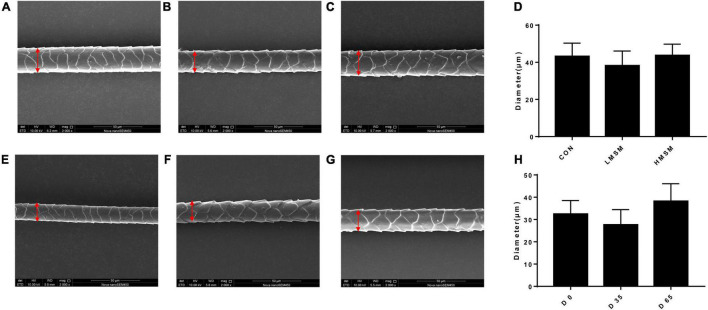
The diameter of hair of kittens fed MSM-supplemented diets. **(A)** The diameter of hair in CON, **(B)** LMSM and **(C)** HMSM on d 65. **(D)** The diameter of hair from kittens fed diets containing MSM on d 65 among three groups. **(E)** The diameter of hair in LMSM on d 0, **(F)** d 35, and **(G)** d 65. **(H)** The diameter of hair from kittens fed diets containing MSM in LMSM on d 0, d 35, and d 65. CON = control diet (0% MSM in diet); LMSM = low dose (0.2% MSM in diet); HMSM = high dose (0.4% MSM in diet). Mean values are based on one kitten per replicate and six replicates per treatment.

A tendency of decreased thickness of scale in the LMSM group compared to the CON group was seen on d 65 (*P* < 0.10; Figures 2A–D). And the scale thickness in the LMSM group changed significantly over time, with the measure on d 65 being lower (*P* < 0.05) than that observed on d 0 (Figures 2E–H). Meanwhile, an increase in the height of scale (*P* < 0.05) in the HMSM group was observed on d 35 compared with that on d 0 and d 65 (Figures 3A–H). No impacts of MSM and time on the hair diameter were identified in the current experiment as shown in Figures 4A–H.

Dietary MSM did not significantly change the hair contents of keratin, S, and C, but enhanced the content of O compared to the CON group (*P* < 0.05, Table 7). Analyses of amino acids in the hair showed that on d 0, Met content in the HMSM group tended to be higher than in the CON and LMSM groups (*P* < 0.10). Besides, Ser content in the CON group tended to be lower than that in the LMSM and HMSM groups (*P* < 0.10), and the content of Trp in the CON group tended to be higher than that in the LMSM and HMSM groups (*P* < 0.10) on d 0. The differences with Met, Ser, and Trp among treatment groups on d 0 were not observed on d 65. Other amino acids did not differ (*P* > 0.05) among dietary treatments on d 0 or d 65.

**TABLE 7:** The contents of S, amino acid, and the keratin in the hair (%) of kittens fed MSM-supplemented diets.

Items	CON	LMSM	HMSM	SEM	*P*-value
**D 0**					
Keratin	73.07	73.88	70.85	1.16	0.608
S	13.97	11.13	12.43	1.24	0.707
C	61.61	59.76	61.10	1.10	0.822
O	19.99	21.80	20.60	0.71	0.638
Met	1.25	1.23	1.06	0.04	0.097
Ser	5.28	5.95	6.01	0.15	0.060
Trp	1.19	0.92	0.76	0.08	0.089
Cys	7.18	7.21	6.62	0.15	0.220
Ala	2.68	2.74	2.76	0.07	0.909
Pro	2.10	5.12	4.22	0.64	0.135
Lys	1.76	2.17	1.90	0.09	0.162
Leu	5.02	6.35	4.85	0.33	0.122
Asp	2.21	2.19	2.16	0.05	0.938
Arg	8.17	8.72	8.40	0.14	0.280
His	30.86	25.23	29.57	1.37	0.230
Gly	14.92	14.72	15.05	0.36	0.946
Thr	3.55	3.74	3.46	0.08	0.370
Glu	5.05	5.28	5.19	0.18	0.896
Val	1.95	1.97	1.76	0.06	0.293
Tyr	3.45	3.40	3.37	0.08	0.934
Ile	1.54	1.40	1.26	0.07	0.333
Phe	1.82	1.66	1.58	0.07	0.348
**D 65**					
Keratin	71.93	71.75	66.05	1.64	0.278
S	11.73	13.40	13.17	0.47	0.340
C	64.32	59.47	59.62	1.44	0.333
O	19.26*^b^*	21.32^a^	20.99^a^	0.37	0.015
Met	1.51	1.37	1.42	0.11	0.902
Ser	8.27	7.73	7.52	0.26	0.550
Trp	1.26	1.38	1.26	0.07	0.766
Cys	5.12	4.82	3.62	0.91	0.820
Ala	3.67	3.25	3.52	0.09	0.181
Pro	6.88	6.17	8.47	0.68	0.425
Lys	3.93	3.36	3.57	0.21	0.597
Leu	7.48	6.58	6.94	0.26	0.421
Asp	5.04	4.04	4.09	0.67	0.835
Arg	10.20	9.88	9.70	0.16	0.510
His	7.90	15.29	12.29	3.00	0.663
Gly	8.98	11.66	11.67	1.33	0.699
Thr	5.47	4.73	4.77	0.34	0.672
Glu	10.69	8.26	9.14	0.98	0.659
Val	3.30	2.89	2.80	0.31	0.823
Tyr	4.88	4.10	4.58	0.20	0.300
Ile	2.44	1.99	2.08	0.22	0.744
Phe	2.98	2.50	2.55	0.30	0.822

*^a,b^Values in a row with no common superscripts differ significantly (P < 0.05).*

*Mean values are based on one kitten per replicate and three replicates per treatment.*

*CON, control diet (0% MSM in diet); LMSM, low dose (0.2% MSM in diet); HMSM, high dose (0.4% MSM in diet).*

### The Fecal Microbiota, Short-Chain Fatty Acids, and Metabolome

**FIGURE 5:**
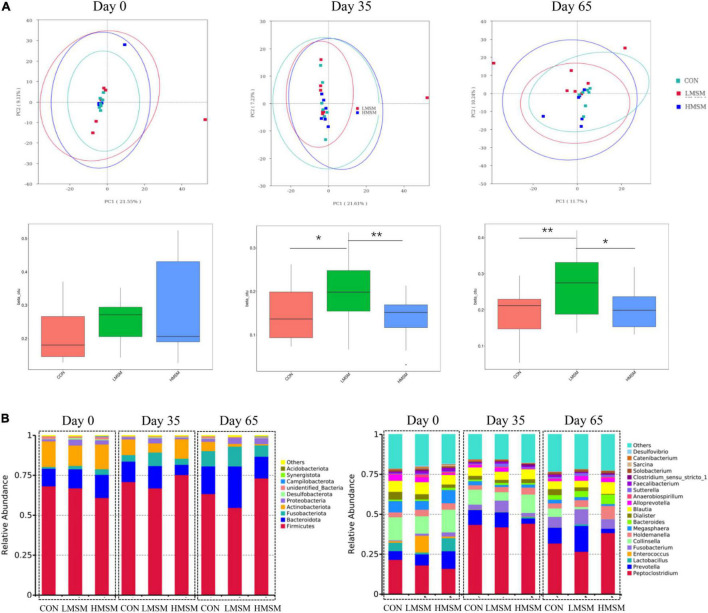
**(A)** Principal coordinate analysis (PCoA) based on weighted UniFrac distances, beta-diversity box plots based on weighted UniFrac distances, **(B)** predominant fecal microbial communities at the phylum and genus levels of kittens fed MSM-supplemented diets on d 0 (CON: *n* = 7; LMSM: *n* = 6; HMSM: *n* = 6), d 35 (CON: *n* = 7; LMSM: *n* = 7; HMSM: *n* = 6), and d 65 (CON: *n* = 7; LMSM: *n* = 7; HMSM: *n* = 6). CON = control diet (0% MSM in diet); LMSM = low dose (0.2% MSM in diet); HMSM = high dose (0.4% MSM in diet). The symbol (*) indicates statistically significant differences between two groups (*P < 0.05 and **P < 0.01).

Assessment of fecal alpha-diversity indices suggested that Goods-coverage and PD-whole-tree tended to be higher in the LMSM group than that in the CON group on d 65 (*P* < 0.10, Table 8). From the difference of beta-diversity index based on weighted UniFrac distances, PCoA plots revealed distinct separation between the CON and LMSM groups on d 35 (*P* < 0.05) and d 65 (*P* < 0.01). Furthermore, the LMSM group showed a significant separation from the HMSM group on d 35 (*P* < 0.01) and d 65 (*P* < 0.05, Figure 5A).

**TABLE 8:** Alpha-diversity indices of fecal microbiota of kittens fed MSM-supplemented diets.

Items	CON	LMSM	HMSM	SEM	*P*-value
**D 0**					
Observed-species	262.00	271.00	259.00	6.20	0.746
Goods-coverage	0.9995	0.9994	0.9994	0.0000	0.778
Shannon	4.88	4.90	4.84	0.10	0.971
PD-whole-tree	19.59	22.79	20.43	1.02	0.443
Simpson	0.91	0.91	0.91	0.01	0.969
Chao1	277.31	286.24	277.05	6.76	0.840
Ace	279.38	289.17	280.42	6.97	0.839
**D 35**					
Observed-species	240.71	258.43	245.83	3.59	0.102
Goods-coverage	0.9994	0.9995	0.9995	0.0000	0.627
Shannon	4.07	4.04	4.14	0.10	0.936
PD-whole-tree	18.21	23.95	18.74	2.02	0.452
Simpson	0.80	0.80	0.80	0.02	1.000
Chao1	258.09	275.01	259.26	4.56	0.238
Ace	259.79	276.87	263.08	4.21	0.208
**D 65**					
Observed-species	286.86	332.86	320.00	11.40	0.230
Goods-coverage	0.9994^a^	0.9991^b^	0.9992^a, b^	0.0000	0.055
Shannon	4.62	4.47	4.44	0.11	0.769
PD-whole-tree	24.54^b^	67.44^a^	41.37^a, b^	7.81	0.060
Simpson	0.86	0.87	0.82	0.02	0.487
Chao1	304.03	356.76	345.22	12.57	0.190
Ace	307.28	362.21	348.86	12.73	0.175

*^a,b^Values in a row with no common superscripts differ significantly (P < 0.05). Mean values are based on one kitten per replicate, seven replicates in CON, and six replicates in LMSM and HMSM on d 0. Mean values are based on one kitten per replicate, seven replicates in CON and LMSM, and six replicates in HMSM on d 35 and d 65.*

*CON, control diet (0% MSM in diet); LMSM, low dose (0.2% MSM in diet); HMSM, high dose (0.4% MSM in diet).*

The predominant fecal phyla of all kittens were Firmicutes, Bacteroidota, Fusobacteriota, Actinobacteriota, Proteobacteria, and Desulfobacterota at various time points (Figure 5B). Furthermore, MSM (whether LMSM or HMSM) caused significant reduction of Actinobacteriota on d 35 (*Q* < 0.05, Table 9). Kittens in the LMSM group had a significantly higher abundance of Proteobacteria compared to kittens in the CON group on d 35 (*Q* < 0.05). The most abundant genera were *Peptoclostridium, Prevotella, Lactobacillus, Enterococcus, Fusobacterium, Collinsella, Holdemanella, Megasphaera, Bacteroides, Dialister*, and *Blautia* at various phases (Figure 5B). Kittens fed MSM displayed significant differences in *Collinsella*, *Subdoligranulum*, *Libanicoccus*, *Methylobacterium-Methylorubrum*, *Sphingomonas*, *Herbaspirillum*, *Ruminococcus-torques-group*, and *Clostridium-sensu-stricto-1* (*P* < 0.05). However, after using the Benjamini–Hochberg test to adjust, there were no significant differences at the genus level (*Q* > 0.05, Table 9).

**TABLE 9:** The differential bacteria at the phylum and genus levels of kittens fed MSM-supplemented diets [based on relative abundance (% of sequences)].

Items	CON	LMSM	HMSM	SEM	*P*-value	*Q*-value		
						
						CON vs. LMSM	CON vs. LMSM	CON vs. LMSM
**D 35**								
**Phylum**								
Proteobacteria	1.36	3.28	1.02	0.32	0.003	0.039	1.000	0.840
Actinobacteriota	9.84	5.72	12.21	0.92	0.007	0.012	1.000	0.013
**Genus**								
*Collinsella*	9.37	5.28	11.53	0.88	0.007	0.179	1.000	0.275
**D 65**								
**Phylum**								
Actinobacteriota	5.83	1.74	0.82	0.86	0.031	0.190	0.160	0.413
**Genus**								
*Collinsella*	5.28	1.41	0.10	0.87	0.031	0.627	0.400	0.644
*Subdoligranulum*	1.12	0.49	0.29	0.12	0.005	0.541	0.104	0.816
*Libanicoccus*	0.11	0.03	0.03	0.01	0.003	0.502	0.166	1.000
*Methylobacterium-Methylorubrum*	0.16	0.43	0.41	0.05	0.014	0.502	0.149	1.000
*Sphingomonas*	0.28	0.71	0.63	0.08	0.037	0.541	0.482	1.000
*Herbaspirillum*	0.09	0.26	0.17	0.03	0.032	0.502	0.617	0.816
*Ruminococcus-torques-group*	0.66	0.53	1.07	0.07	0.004	0.804	0.697	0.644
*Clostridium-sensu-stricto-1*	0.24	0.28	0.70	0.06	0.002	1.000	0.718	0.732

*Mean values are based on one kitten per replicate, seven replicates in CON, and six replicates in LMSM and HMSM on d 0. Mean values are based on one kitten per replicate, seven replicates in CON and LMSM, and six replicates in HMSM on d 35 and d 65.*

*CON, control diet (0% MSM in diet); LMSM, low dose (0.2% MSM in diet); HMSM, high dose (0.4% MSM in diet).*

**FIGURE 6:**
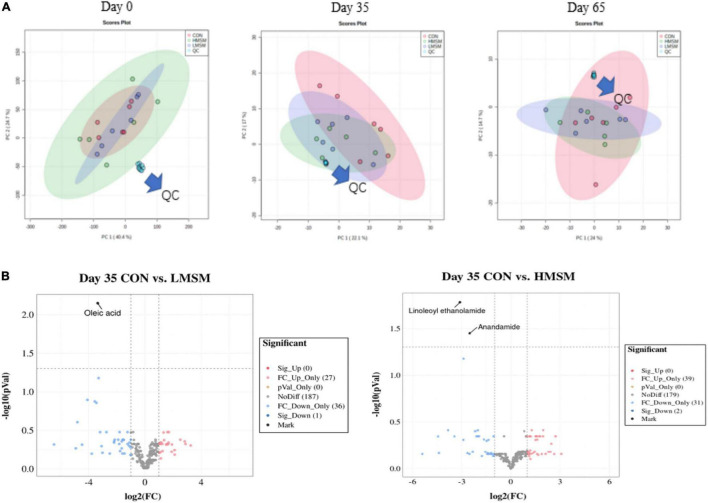
**(A)** Score plots from the PCA model among three groups on d 0, d 35, and d 65, **(B)** the predominant metabolites on d 35 (CON: *n* = 7; LMSM: *n* = 7; HMSM: *n* = 6). CON = control diet (0% MSM in diet); LMSM = low dose (0.2% MSM in diet); HMSM = high dose (0.4% MSM in diet).

The PCA score plots showed no obvious separations among the groups at varying time points (Figure 6A). In this study, a total of 260 metabolites were detected at all stages. The changes in metabolites over time were shown in Figure 6B. By screening differential metabolites in the MSM groups, fecal oleic acid, linoleoyl ethanolamide, and anandamide were found to be downregulated on d 35. In addition, the metabolites had no significant differences on d 65. No significant differences in SCFAs and BCFAs were observed among the three groups (*P* > 0.05, Table 10).

**TABLE 10:** SCFAs content of fecal samples of kittens fed MSM-supplemented diets.

Items	CON	LMSM	HMSM	SEM	*P*-value
**D 0**					
Total SCFAs, (μg/g)	2981.42	2722.26	2829.12	88.12	0.511
Acetic acid, (μg/g)	1455.18	1325.22	1430.48	44.02	0.468
Propionic acid, (μg/g)	984.77	884.83	914.75	40.11	0.608
Butyric acid, (μg/g)	541.47	512.22	483.88	17.93	0.449
Total BCFAs, (μg/g)	679.06	515.81	481.68	49.33	0.227
Isobutyric acid, (μg/g)	142.24	89.93	88.87	13.74	0.199
Isovaleric acid, (μg/g)	223.10	145.98	147.50	19.42	0.184
Valeric acid, (μg/g)	313.72	279.90	245.31	18.21	0.327
**D 35**					
Total SCFAs, (μg/g)	2605.18	2761.29	2631.05	79.20	0.715
Acetic acid, (μg/g)	1330.84	1405.21	1335.60	41.28	0.737
Propionic acid, (μg/g)	748.95	815.98	759.28	45.65	0.830
Butyric acid, (μg/g)	525.38	540.11	536.17	11.92	0.886
Total BCFAs, (μg/g)	779.08	773.44	820.79	39.17	0.879
Isobutyric acid, (μg/g)	187.54	179.52	205.70	14.78	0.782
Isovaleric acid, (μg/g)	275.92	277.04	316.07	15.84	0.527
Valeric acid, (μg/g)	315.62	316.88	299.03	14.03	0.861
**D 65**					
Total SCFAs, (μg/g)	2775.90	2867.60	2659.24	60.54	0.394
Acetic acid, (μg/g)	1429.65	1453.76	1447.88	22.75	0.914
Propionic acid, (μg/g)	820.82	854.43	691.23	40.55	0.232
Butyric acid, (μg/g)	525.43	559.41	520.12	11.93	0.367
Total BCFAs, (μg/g)	825.05	804.35	719.67	43.70	0.609
Isobutyric acid, (μg/g)	190.47	199.52	172.81	15.38	0.792
Isovaleric acid, (μg/g)	296.77	300.98	272.88	14.57	0.722
Valeric acid, (μg/g)	337.81	303.86	273.98	17.83	0.365

*Mean values are based on one kitten per replicate, seven replicates in CON, and six replicates in LMSM and HMSM on d 0.*

*Mean values are based on one kitten per replicate, seven replicates in CON and LMSM, and six replicates in HMSM on d 35 and d 65.*

*CON, control diet (0% MSM in diet); LMSM, low dose (0.2% MSM in diet); HMSM, high dose (0.4% MSM in diet).*

*Total SCFAs (short-chain fatty acids) = acetate acid + propionate acid + butyrate acid; Total BCFAs (branched-chain fatty acids) = isobutyrate acid + isovalerate acid + valerate acid.*

## Discussion

Methylsulfonylmethane may serve as functional ingredients in the pet diet due to its beneficial effects on hair quality and influence on gut microbiota and metabolomics. In the present study, we selected Ragdoll Kittens as model animals to evaluate the effects of dietary supplementation with different levels of MSM on physiological function, antioxidant capacity, hair quality, microbiota, and metabolomics. As expected, all the three experimental diets had the same ingredient composition and nutrient levels, except for the supplementation of 0.2% or 0.4% MSM. Briefly, kittens fed with MSM had no effect on growth performance, serum biochemical parameters, antioxidant capacity, fecal microbiota, and metabolic profiles, while supplementation with MSM improved hair quality. To the best of our knowledge, this is the first study to investigate the effect of MSM supplementation in cats.

Hair growth in the HMSM group was faster than the CON and LMSM groups from d 0 to d 35, but slowed down from d 36 to d 65. The situation was the opposite in the LMSM group, where hair growth was slower from d 0 to d 35 but developed faster from d 36 to d 65 than in the other two groups. Despite not showing the hair growth effect as good as HMSM early during the experiment, LMSM was more effective for continuous hair growth. The hair score results showed no significant differences among the treatment groups. However, the hair in the LMSM groups was evaluated as more ideal than other groups, and the hair in the CON group was greasier than in the MSM groups.

Scale structure is an important aspect for evaluating hair quality (Mishra et al., 2016). The thickness, density, shape, and arrangement structure of the scale can directly affect the physical and chemical properties of the hair, such as glossiness, softness, and feel. A thinner scale corresponds to a smaller friction coefficient and a smoother scale surface. In the case of greater scale height, the number of scales per unit length will be fewer, and the scale density will be smaller, resulting in smoother hair. If the hair diameter is larger, the softness of the hair will be worse (Wei et al., 2005; Liu et al., 2017). Given that the scale thickness in the LMSM group was smaller than that in other groups and decreased over time, the hair of kittens in the LMSM group became smoother over the experimental period. The scale height in the HMSM group on d 35 was higher than that on d 0 and d 65, indicating that the hair in this group got smoother during the period from d 0 to d 35, but this positive effect was retrieved at the end of d 65. There were no significant differences in the diameter of the hair among the treatment groups. To sum up, dietary MSM has beneficial effects on hair quality, making hair smoother and glossier. A high dose of MSM (0.4%) fits the requirement for short-term use, and a low dose (0.2%) is more suitable for long-term inclusion.

To further investigate the mechanism of MSM supplementation on hair quality, the content of elements, sulfur-containing amino acids, and keratin were measured. It has been proposed that the nourishing effects of MSM on skin, hair, and fingernails are due to its high sulfur content (Richmond, 1986). However, there are no significant effects on the content of S on d 0 and d 65 in our study. A relatively high concentration of [^35^S] MSM was detected in the blood, spleen, and hair tissues, and the administered MSM might have been metabolized to yield certain sulfur-containing compounds, such as keratin, a key component of hair and nail in rats (Otsuki et al., 2002). The study also discusses the relationships between the amounts of MSM and Met, and the results show that there is no clear evidence to support the hypothesis that MSM may be metabolized to generate methionine or its metabolites. In agreement with their results, dietary MSM also did not change the hair content of Met in our study. More research is needed in the future to determine the metabolic mechanisms of MSM effects on hair quality in kittens.

The gut is host to a diverse and abundant community of bacteria that influence health and disease susceptibility. The gut microbiota contributes to host metabolism and protects against pathogens, thereby affecting directly or indirectly host physiological functions (Pilla and Suchodolski, 2020). Our results showed that MSM had no negative effect on fecal microbiota. Dietary supplementation with LMSM increased bacterial diversity. Our results also showed that MSM markedly inhibited the growth of Actinobacteria on d 35. Actinobacteria were shown to be increased in dogs with chronic enteropathies compared to healthy control dogs (Allenspach et al., 2010; Suchodolski et al., 2012). These data suggest that a decrease in fecal Actinobacteria is potentially beneficial for the animal, but more research is needed to confirm this suggestion. The genus *Collinsella* belongs to the family Coriobacteriaceae and the phylum Actinobacteria. Members of the family Coriobacteriaceae are considered as pathobionts, and *Collinsella* is its dominant taxon (Chow et al., 2011),the abundance of which has been associated with type-2 diabetes (Lambeth et al., 2015), rheumatoid arthritis (Chen et al., 2016), and cholesterol metabolism (Ridlon et al., 2006). In addition, gnotobiotic approaches have shown that administration of *Collinsella* reduces the expression of tight junction proteins in enterocytes and stimulates gut leakage (Chen et al., 2016). Currently, the molecular mechanisms by which *Collinsella* affects host metabolism are unknown. A lower relative abundance of *Collinsella* was noticed in fecal samples of kittens fed MSM after 65 d, which may exert a beneficial effect on maintaining host intestinal health. The genus *Subdoligranulum* was less abundant in humans (Bajaj et al., 2012; Qin et al., 2014). Leclercq et al. (2014) observed that the *Subdoligranulum* genus was significantly more abundant in patients with poor gut integrity and high gut permeability (Leclercq et al., 2014). *Lachnospiraceae* exhibited higher relative abundance in patients with inflammatory bowel disease (IBD) and further led to severe colitis (Quraishi et al., 2017), and the *Lachnospiraceae_NK4A136_group* is the main genus of Lachnospiraceae. The increase in the abundance of *Lachnospiraceae_NK4A136_group* has been demonstrated in mouse models of colitis and diet-induced obesity, suggesting pathological roles for these bacteria in disease-associated inflammation (Liu et al., 2019; Shao et al., 2020). Furthermore, Zhang et al. (2021) reported that fat metabolism positively correlated with the relative abundance of *Sphingomonas*, which is more abundant in high body weight individuals (Zhang et al., 2021). Another study reported that sphingolipids from *Sphingomonas* could control different cellular processes, including migration, apoptosis, and proliferation (Bryan et al., 2016). For instance, *Sphingomonas* was shown to synthesize sphingosine and later formed sphingomyelin with fatty acids derivatives, thus reducing fat deposition in the chicken liver. These findings suggested that the abundance of the *Sphingomonas* genus was significantly correlated with hepatic fat metabolism (Li et al., 2020). Thus, our results showed that MSM had no negative impact on the gut microbes of kittens. Further research is needed to provide a clear explanation.

Microbial-derived metabolites often are secreted in the intestine and translocated across the intestinal barrier into the circulating system and serve as important modulators for host metabolism. In our study, metabolomics based on UPLC-Orbitrap-MS/MS analysis method was applied to investigate the changes in fecal metabolites. Screening of differential metabolites in the MSM groups revealed that MSM only downregulated oleic acid, linoleoyl ethanolamide, and anandamide on d 35. More important is that there are no differential metabolites on d 65, which means the dietary supplementation with MSM had no negative effect on metabolism. Due to the lack of studies on the mechanism of MSM metabolism, further research is needed to provide a clear explanation between MSM and fecal metabolome in kittens supplemented with dietary MSM.

Short-chain fatty acids are the end products of the fermentation of dietary fibers by the anaerobic intestinal microbiota, which have been shown to exert multiple beneficial effects on mammalian energy metabolism (Besten et al., 2013). In our study, MSM did not exert an effect on fecal SCFAs. Taken together, MSM has no detrimental influence on the gut microbiota composition and its metabolites.

## Conclusion

Dietary supplementation with MSM had no effect on growth performance. The analysis of the hair data indicated that MSM made hair smoother and glossier, suggesting potential benefits to the hair quality of kittens. The antioxidant capacity and serum biochemistry were not altered by MSM, which supports the safety of MSM inclusion to a certain degree in feline diets. The microbiota and metabolomic analyses revealed that MSM has no detrimental influence on the gastrointestinal health of kittens. In summary, we systematically elucidated the beneficial effects of MSM treatment, particularly 0.2% MSM, on kittens, but more research is needed to systematically elucidate it.

## Data Availability Statement

The datasets presented in this study can be found in online repositories. The names of the repository/repositories and accession number(s) can be found below: https://www.ncbi.nlm.nih.gov/, PRJNA843192.

## Ethics Statement

The animal study was reviewed and approved by the Animal Care and Use Committee; the Laboratory Animal Center at the South China Agricultural University.

## Author Contributions

DG and LMZ were responsible for animal care, breeding, and sampling. DG contributed to the data analysis and drafting of the manuscript. LNZ, SH, and KY contributed to the critical revision of the manuscript. KY made feasible suggestions for the manuscript. XL and CW contributed to the sample analysis. AT and MZ contributed to the data analysis. YY and BD contributed to the conception, experimental design, and data interpretation. All authors contributed to the article and approved the submitted version.

## Conflict of Interest

AT was employed by Guangzhou Qingke Biotechnology Co., Ltd. The remaining authors declare that the research was conducted in the absence of any commercial or financial relationships that could be construed as a potential conflict of interest.

## Publisher’s Note

All claims expressed in this article are solely those of the authors and do not necessarily represent those of their affiliated organizations, or those of the publisher, the editors and the reviewers. Any product that may be evaluated in this article, or claim that may be made by its manufacturer, is not guaranteed or endorsed by the publisher.

## Footnotes

1
https://www.metaboanalyst.ca
